# Fitting in or standing out: a scoping review of gender-related findings in Nordic EMS through an organizational socialization lens

**DOI:** 10.1186/s13049-026-01677-3

**Published:** 2026-08-17

**Authors:** Lindström Veronica, R. C. Ericsson, R. Sinisalo, A. Venesoja

**Affiliations:** 1 Department of Nursing, Division of Ambulance Service, Region Västerbotten, Umeå University, Umeå, Sweden; 2 School of Business and Healthcare, Arcada University of Applied Sciences, Helsinki, Finland; 3 LUT University, Lappeenranta, Finland; 4 LAB University of Applied Sciences, Lappeenranta, Finland; 5 Department of Emergency Care and Services, University of Helsinki, Helsinki University Hospital, Helsinki, Finland

## Abstract

**Objective:**

To explore and map research on workplace culture, occupational health, exposure to violence, and organizational dynamics among Emergency Medical Service (EMS) professionals in Nordic countries, focusing on studies reporting gender-related findings between 2010 and 2025.

**Methods:**

A scoping review was conducted in accordance with the JBI methodological guidance and reported using the PRISMA-ScR framework. Searches were performed in CINAHL, PubMed, Scopus, Web of Science, and Google Scholar covering the period 2010–2025. Studies focusing on EMS professionals and workplace experiences related to gender, workplace culture, discrimination, leadership, or organizational factors were eligible. Data were charted descriptively, using an inductive content analysis approach.

**Results:**

Seven peer-reviewed studies and five master’s theses met the inclusion criteria, comprising cross-sectional, prospective cohort, and observational designs. Three explicitly examined gender/equality; other gender-related differentiated findings were reported as secondary observations. Women reported higher physical and psychosocial work demands (43% vs. 22%; 41% vs. 20%), greater concerns about threats and violence (58% vs. 38%), and higher levels of mental health problems (29% vs. 8%) and sleep disturbances (60% vs. 39%). Despite this, women demonstrated higher physical capacity, while men had higher cardiovascular disease risk factors. Men reported greater exposure to certain critical incidents, whereas stress levels were similar between genders. Workplace violence was common, with differences between men and women observed in exposure, perception, and support-seeking behaviors.

**Conclusion:**

This scoping review found that gender-related patterns are present across workplace demands, health outcomes, exposure to violence, and critical incidents, but are rarely explicitly addressed in Nordic EMS research. Rather than isolated findings, these patterns appear to be embedded in organizational processes that shape norms, expectations, and professional identities. For example, women reported higher work demands, greater concern about workplace risks, and poorer mental health outcomes, despite demonstrating high physical capacity, while men reported greater exposure to certain critical incidents. Interpreted through the lens of organizational socialization, these findings suggest unequal access to legitimacy, well-being, and career opportunities, with implications for workforce sustainability. Addressing these issues through organizational and educational strategies may improve working conditions, reduce workplace risks, and support a more equitable and sustainable Nordic EMS workforce.

**Supplementary Information:**

The online version contains supplementary material available at 10.1186/s13049-026-01677-3.

## Background

Emergency Medical Services (EMS) professionals work in complex and unpredictable environments in which organizational structures, professional culture, and workforce composition shape well-being, performance, and retention [1–4]. Historically, EMS has developed as a male-dominated field, influenced by organizational cultures resembling those of other uniformed services such as police, fire services, and the military [5–7]. These cultures are often described as performance-oriented, stoic, and resistant to change, reflecting a “heroic” or endurance-based professional ideal [8].

Although the proportion of women entering EMS education and practice has increased internationally [9], similar trends have been observed in Nordic countries, yet systematic data remain limited [10]. However, numerical change does not necessarily indicate a corresponding transformation in workplace culture. Evidence from outside the Nordic region suggests that gender-based inequalities persist, particularly in leadership representation [11–13]. This discrepancy points toward a mismatch between increasing workforce diversity and the organizational norms that continue to shape professional roles, expectations, and opportunities. Similar patterns have been described across male-dominated occupations more broadly, where organizational cultures and gendered working environments have been shown to shape workplace experiences and contribute to persistent gender inequalities despite increasing workforce diversity [14, 15]. Such dynamics have been described as tokenism, where individuals in numerical minorities are expected to adapt to dominant norms rather than reshape them [16]. However, Williams demonstrated that these dynamics are not symmetrical, showing that men in minority positions may experience a “glass escalator,” benefiting from accelerated career progression. This suggests that minority status does not operate uniformly, but is mediated by gendered power relations, whereby men may experience advantages such as accelerated career progression, whereas women more often encounter barriers [17].

One way to understand how these gender-related patterns are reproduced is through the lens of organizational socialization. Organizational socialization refers to how individuals develop role clarity, task competence, and social integration within a professional community [18]. For EMS professionals, this involves not only acquiring clinical competence but also learning implicit norms related to behavior, communication, emotional expression, and professional identity [2–4, 19, 20]. Such structures can be understood as gendered, as organizational practices, norms, and expectations are not neutral but embedded in broader gendered systems of meaning and power [15]. Expectations formed prior to entering the profession may already reflect gendered assumptions, while onboarding ranges from structured approaches to informal learning through peer interaction. In environments shaped by norms such as toughness, emotional control, and performance under pressure, fitting in may require adaptation to expectations that do not equally reflect all workers’ identities [8, 21]. This creates tension between “fitting in” and “standing out” [22].

Socialization processes can further be understood through role clarity, social integration, and task mastery [18]. These dimensions are central for professional functioning but may not be equally accessible. In gendered organizational contexts, expectations regarding behavior and competence may shape access to inclusion, learning opportunities, and recognition [15, 22]. Inclusion is closely linked to psychological safety in healthcare settings, enabling individuals to speak up and engage in learning without fear of negative consequences [23, 24]. While social integration has been linked to professional development and occupational risk, it remains unclear how key socialization processes, such as role clarity, integration, and task mastery, operate in relation to gender within Nordic EMS. Consequently, gendered organizational dynamics have received limited theoretical attention, constraining current understandings of how gendered experiences are shaped within EMS organizations. This gap is particularly relevant, as organizational socialization theory suggests that mechanisms such as anticipatory expectations, socialization tactics, and differential access to role clarity and social integration are likely to shape workplace experiences and outcomes [18, 22].

Understanding how gender inequalities manifest within EMS workplaces is important for workforce sustainability, professional well-being, leadership diversity, and safe working environments [13]. It may also have implications for patient care, as workplace culture, professional well-being, and team functioning are known to influence patient safety and quality of care [25]. Nevertheless, in the Nordic context, where gender equality is often assumed to be well established [26], identifying potential structural inequalities remains particularly relevant. Therefore, the review question becomes: What evidence exists regarding workplace culture, occupational health, exposure to violence, and organizational dynamics among EMS professionals in Nordic countries, and how are findings reported for women and men?

## Methods

The objective was to explore and map research on workplace culture, occupational health, exposure to violence, and organizational dynamics among EMS professionals in Nordic countries, focusing on studies reporting gender-related findings between 2010 and 2025. This study was conducted as a scoping review following methodological guidance from the JBI [27, 28] and reported in accordance with PRISMA-ScR [29], see Supplementary File 1. A scoping review was considered the most appropriate methodology because it enables the systematic mapping of the extent, range, and characteristics of evidence within broad and heterogeneous fields of research, while also identifying knowledge gaps. A study protocol was published online prior to the start of the study (https://osf.io/2gw6s/overview).

### Search strategy

Guided by a senior librarian (RS), searches were conducted in Medline, Scopus, and Web of Science. The complete search strategies for Medline, Scopus, and Web of Science are provided in Supplementary File 2. The search strategy was structured according to the Population–Concept–Context (PCC) framework, where the population comprised EMS professionals, the concept addressed gender-related workplace dynamics (e.g., gender inequality, workplace culture, discrimination, leadership, and organizational dynamics), and the context was EMS organizations in the Nordic region (see Table 1). Furthermore, to ensure comprehensive identification of relevant literature, the search strategy also included broader European search terms and studies conducted in Europe, as these could include data from one or more Nordic countries. Eligibility was subsequently determined during study selection based on whether Nordic data were reported. The search strategy combined controlled vocabulary and free-text terms related to the population and concept. Searches were limited to peer-reviewed publications between January 2010 and November 2025 and to languages accessible to the research team (English, Finnish, and Swedish). The final search in the scientific databases was conducted on 28 November 2025. To enhance comprehensiveness, supplementary search strategies were applied, including manual screening of reference lists. Furthermore, searches in Google Scholar were conducted to identify grey literature, given the limited number of peer-reviewed publications. The Google Scholar search included combinations of Swedish and Finnish search terms related to EMS and gender. The final Google Scholar search was conducted on 25 April 2026. The purpose of these searches was to capture potentially relevant academic work not indexed in the selected databases.

**Table 1:** Population–Concept–Context (PCC) framework used

Population:	Concept:	Context:
*EMS professionals working in Nordic countries*	*Gender differences related to Workplace culture*,* occupational health*,* exposure to violence*,* and organizational dynamics*	*Emergency Medical Services in the Nordic countries*

### Eligibility criteria

Criteria for study inclusion were that they involved EMS professionals (e.g., nurses, ambulance personnel, paramedics, or other prehospital emergency care clinicians) and addressed workplace experiences, organizational culture, occupational health, or exposure to violence. Studies were included if they reported gender-related findings, defined as results presented separately for women and men or through explicit analysis of gender differences, and were conducted in Nordic contexts or included Nordic EMS populations. Studies of qualitative, quantitative, or mixed-methods design were eligible for inclusion. Studies published in English, Finnish, and Swedish were eligible, and, to capture relevant grey literature, theses at a minimum of master’s level were also considered. As scoping reviews aim to comprehensively map available evidence, master’s theses were considered eligible when they met the same inclusion criteria as peer-reviewed studies. Studies focusing exclusively on patient outcomes without reference to EMS professionals were excluded. Conference abstracts, editorials, and other non-peer-reviewed publications were also excluded. In cases where studies included broader populations, only those with extractable Nordic EMS data were considered eligible.

### Study selection and data extraction

**Fig. 1:**
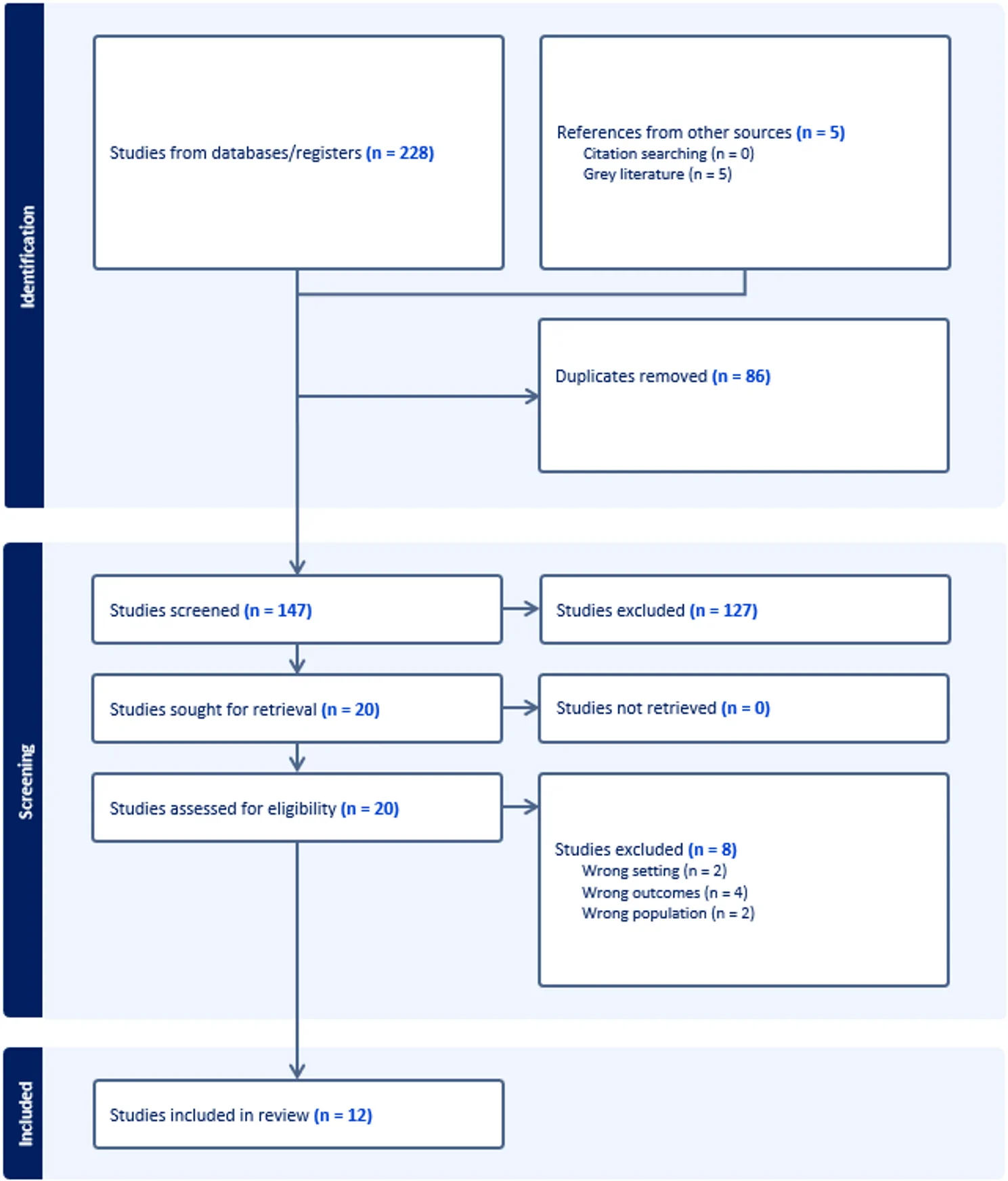
PRISMA flow diagram of the study selection process for studies on gender-related findings in Nordic Emergency Medical Services

All references were imported into Covidence, where automatic duplicate detection was performed, followed by manual verification prior to screening. Thereafter, two reviewers (CE & AV) independently screened titles and abstracts for potential eligibility (*n* = 147), followed by full-text assessment of studies meeting the inclusion criteria (*n* = 20) as displayed in the PRISMA Flowchart (Fig. 1). Reasons for exclusion at the full-text stage were documented and categorized according to the primary reason for exclusion (e.g., non-EMS population, non-eligible setting, absence of gender-related findings, or outcomes not relevant to the question and aim). Disagreements at any stage of the screening process were resolved through discussion, and when necessary, in consultation with the co-author (VL) to reach a consensus. Data extraction was performed collaboratively by VL, CF and AV using a structured charting approach. CE and VL extracted data from the English-language studies, whereas CE and AV extracted data from the Finnish-language master’s theses and CE and VL from the Swedish-language master’s theses. Extracted information included study characteristics (e.g., authors, year of publication, country, study design, study population, and whether gender was included in the study main aim) together with findings relevant to the review objective. As gender was often reported as a secondary or incidental finding rather than the primary focus of the included studies, VL, CF and AV discussed these findings throughout the charting process to reach consensus regarding their relevance and how they should be extracted and categorized in relation to the review question.

### Analysis

The analysis was conducted using a descriptive and inductive approach and was not guided by organizational socialization theory. Rather, organizational socialization was applied subsequently as an interpretive lens to support the discussion of the findings. Following charting, two authors (VL and CE) synthesized the extracted findings into categories based on similarities in reported outcomes, and a third author (AV) reviewed and verified the categorization. The categorization process was iterative and involved grouping findings related to workplace demands and health outcomes; exposure to workplace violence and gender discrimination; critical incidents; stress, support-seeking, and professional roles; and competence. The aim of the analysis was not to generate new theoretical interpretations but to map and synthesize how gender-related findings were reported across studies. Patterns across studies were identified and summarized within each category. Grey literature was synthesized alongside peer-reviewed studies to provide a comprehensive mapping of the available evidence.

## Results

The seven peer-reviewed studies and the five master’s theses included cross-sectional surveys, prospective cohort studies, quantitative observational designs, and one qualitative interview study conducted in Nordic EMS contexts. Studies from Sweden, Norway, Finland, and Denmark were represented, with the majority originating from Sweden and Finland. Five studies explicitly addressed gender or gender inequality, while the remaining studies reported gender-related findings as secondary observations within broader research on workplace conditions, occupational health, violence, and exposure to critical incidents, as displayed in Table 2.

**Table 2:** Descriptions included studies, theses, and summaries of findings

Author, Year, Country	Aim(s)	Gender as primary study aim (Yes/No)	Study design	Population and sample	Key findings
Chappatte Jonsson, R. & Lindberg, J. (2025) Sweden (Master Thesis)	To highlight female ambulance nurses’ experiences of being subjected to harassment in their professional role.	Yes	Qualitative	Eight female ambulance nurses	Female ambulance nurses experienced gender-based discrimination, gendered workplace norms, and limited organizational support, resulting in emotional distress, pressure to demonstrate competence, and reliance on informal support networks.
Jansson et al. (2020) Sweden	To investigate and compare self-reported professional competence among nurses working in the ambulance service and to explore associations between potentially predictive background factors and self-reported professional competence.	No	Cross-sectional	Three ambulance organizations located in central Sweden. A total of 105 nurses participated by responding to a digital questionnaire, resulting in a response rate of 21%.	Gender differences in self-reported competence were observed: women scored higher in Nursing Care (*p* = 0.028) and Value-Based Nursing (*p* = 0.047), while men scored higher in Medical Technical Care (*p* = 0.048) and Care Environments in Serious Events (*p* = 0.011).
Johnsen et al. (2024), Sweden	To describe work-, lifestyle-, and health-related factors among ambulance personnel, and to analyze differences between women and men.	Yes	Cross-sectional	106 ambulance clinicians self-reported and objective measures of work, lifestyle, and health in 10 Swedish ambulance stations	Compared with men, women reported higher physical (43% vs. 22%) and psychosocial (41% vs. 20%) work demands, more awkward working postures (77% vs. 55%), greater concerns about violence (58% vs. 38%) and exposure to threats (66% vs. 37%), more mental health problems (29% vs. 8%), and more sleep disturbances/worry (60% vs. 39%). Women performed better in several physical capacity tests and had lower cardiovascular disease (CVD) risk, whereas men had more CVD risk factors.
Juliusdatter Haug et al. (2025) Norway	To adapt and validate the Staff Observation Aggression Scale – Revised (SOAS-R) for use in ambulance services (SOAS-RA), and to examine the relationship between SOAS-RA severity scores and staff’s subjective perceptions of incident severity using a Visual Analogue Scale (VAS)	No	Delphi	402 reports from ambulance professionals in 34 ambulance stations	Female ambulance staff rated incidents of workplace violence as more severe than male staff, despite no differences in objectively measured severity scores, indicating potential gender differences in perception and appraisal of workplace threats.
Kettunen, M. (2022), Finland (Master Thesis)	To examine the current state of equality in the North Karelia Rescue Service and prepare an equality plan for them & to produce concrete action proposals to facilitate equal practises and improve employees’ experiences of equality.	No	Multi-method (literature review and survey)	One rescue department. Participants: *n* = 174. (Paramedics: 34% *n* = 59)	Inappropriate behavior was reported by 47% of respondents, most commonly related to age (24%) and gender (21%). Discrimination was perceived to occur by 35% of respondents, while 21% reported personal experiences of discrimination. Gender was the most frequently identified reason for discrimination (27%).
Koskela A. (2025) Finland (Master Thesis)	The aim was to analyze the relationships between background variables, internal (confidence, expected traits of a leader), and external factors (perceived equality, double standards, influence of parenthood) and paramedics’ perceptions of becoming an EMS field commander.	No	Cross-sectional	Paramedics in four Finnish rescue departments providing EMS and EMS field management (*n* = 632) during spring 2025.	Although women (*n* = 324) and men (*n* = 308) were equally represented among paramedics, all Emergency Medical Service (EMS) field commanders were men (*n* = 32). Men applied for leadership positions twice as often as women despite equal interest. Women reported less favorable perceptions of equality, and longer work experience was associated with applying for field commander roles among women (*p* = 0.027) but not men (*p* = 0.174).
Luoma, K. (2020) Finland (Master Thesis)	To identify the current state of experiences in equality and to promote equality through behavioral economics in the field of rescue services.	No	Multi-method (2 surveys)	Eleven rescue services (including paramedics) in Finland. Participants: *n* = 337, response rate 15%.	Fewer than 23% of all respondents reported personally experiencing gender discrimination; the burden was strongly concentrated among women: nearly 69% of women had experienced gender-based discrimination, and as many as 85% had observed it. The burden was most pronounced in prehospital emergency care, where younger women at the beginning of their careers appeared especially vulnerable, whereas discrimination was reported less often in expert and accident prevention roles with more balanced gender representation.
Nordqvist et al. (2023) Finland	To examine: (1) the prevalence of Critical incidents (CI) experiences among Finnish women and men paramedics; (2) the amount of stress related to the CIs; (3) the association between CI-S and intention to leave the profession; and (4) the needed and received support for CI-S.	Yes	Cross-sectional	427 Paramedics from 8 Finnish ambulance organizations answered a survey	Men reported significantly greater exposure to several types of critical incidents, including death-related, child-related, burn, and suicide cases (*p* = 0.010 to *p* < 0.001), whereas CI-related stress levels were similar between women and men (20.21 vs. 19.70). Women were more likely to seek healthcare support, while colleagues were the main support source for both genders.
Petzäll et al. (2011) Sweden	Aim was to investigate the incidents of threats and violence within the Swedish ambulance service and to describe these situations	No	Cross-sectional	134 registered nurses and paramedics from 11 ambulance stations answered a survey	Women reported higher exposure to threats (35% vs. 21%) and physical violence (23% vs. 12%) than men, although differences were not statistically significant. Overall, 66% reported lifetime exposure to threats or violence, leading to increased caution in patient encounters.
Schøsler et al. (2024) Denmark	To describe the extent of and subsequent reporting of physical and psychological workplace violence toward the prehospital healthcare workers in Denmark in a two-year period & to elucidate any possible effect of workplace violence on the private and professional lives of the prehospital healthcare personnel.	No	Cross-sectional	584 prehospital health care workers answered a survey from different parts of Denmark	Although no gender-specific statistical comparisons were reported, women appeared proportionally more represented among those reporting psychological violence, whereas reported physical violence was similar in relative terms. In absolute numbers, most exposed respondents were men, reflecting the male-dominated sample (82.7%).
Viking et al. (2024) Sweden	To measure the 1-year cumulative incidence of and analyse the risk factors associated with workplace violence directed towards the ambulance service in a Swedish region	No	Prospective cohort study	28 640 ambulance assignments in one region in Sweden	Women reported higher exposure to threats (35% vs. 21%) and physical violence (23% vs. 12%) than men; however, no statistically significant gender differences were found. Overall, 66% reported lifetime exposure to threats or violence.
Ylönen, J. (2019), Finland (Master Thesis)	The aim of the study was to find out how common the sexual harassment experienced by the primary care community in the Central Rescue Department of Finland is, and what factors expose paramedics and students to sexual harassment.	No	Cross-sectional	One rescue department and one University of Applied Sciences. Target groups: rescue workers, paramedics, paramedic students. Participants: *n* = 99, response rate 24%.	Overall, 33% of respondents reported experiencing sexual harassment. Women reported sexual harassment markedly more often than men (53% vs. 18%). Harassment was most perpetrated by patients/clients (84%) and colleagues (57%), with 94% of incidents involving a harasser of the opposite sex.

### Workplace demands and health outcomes

Only one study specifically examined gender differences in workplace demands and health outcomes. The study findings showed higher physical and psychosocial work demands among women, together with poorer mental health outcomes, despite the fact that women demonstrated higher objective physical capacity than men [30]. Specifically, women reported higher physical and psychosocial work demands than men. Specifically, women reported higher physical work demands (43% vs. 22%), more frequent exposure to awkward or bending working positions (77% vs. 55%), and higher psychosocial work demands (41% vs. 20%) [30]. In terms of health outcomes, women reported higher levels of mental health problems (29% vs. 8%) and more frequent sleep disturbances and worry (60% vs. 39%). Furthermore, women showed a higher intention to leave EMS work (28.2% vs. 16.0%). Despite these findings, women performed better than men on several objective tests of aerobic capacity, hand strength, and flexibility. The authors suggest that this high physical capacity may be a prerequisite for women to manage the inherent physical demands of the role. Men had higher levels of cardiovascular disease risk factors, while women showed a lower risk of cardiovascular disease [30].

### Workplace violence and gender discrimination exposure

Across three quantitative studies, exposure to threats and violence emerged as a recurring finding. Although the measures differed, a consistent pattern was that women reported greater concern about violence and higher exposure than men [30–32].

Petzall et al. and Viking et al. showed that threats and violence were common among EMS professionals, with 66% reporting lifetime exposure and 47.4% and 25.7% reporting recent psychological and physical violence, respectively [31, 32]. Threats and violence were frequently underreported and linked to negative psychological and work-related outcomes [33]. Threats were mainly verbal, but 27% involved weapons, and physical violence typically involved pushing, hitting, or kicking [32]. Women rated aggressive incidents as more severe than men, despite similar self-reported severity scores [34]. Women reported greater concerns about threats and violence compared to men (58% vs. 38%), and a higher proportion reported having been subjected to threats (66% vs. 37%) [30]. Consistent findings were reported by Petzall et al., where women reported higher exposure to threats (35% vs. 21%) and physical violence (23% vs. 12%) compared to men, although these differences were not statistically significant [32]. Furthermore, Viking et al. showed that women reported significantly more frequent exposure to threats (66% vs. 37%). However, violence was frequently underreported, with 60.7% not reporting physical violence and 83.0% not reporting psychological violence to their employer. The most common reasons for not reporting were that incidents were perceived as not serious enough or that reporting was unlikely to make a difference [33].

Evidence regarding discrimination, harassment, equality and leadership originated primarily from three master’s theses [35–37]. Although conducted in different EMS and rescue service settings, they consistently described gender-related discrimination, harassment and organizational cultures that disproportionately affected women. Luoma reported that fewer than one-quarter of respondents had personally experienced gender discrimination, but these experiences were concentrated among women: nearly 69% of female respondents reported experiencing gender-based discrimination, and 85% observed it. Gender-based discrimination appeared particularly pronounced among younger female EMS professional in the early stages of their careers [35]. Ylönen further showed that harassment was a gendered manifestation of inappropriate behavior in EMS work environments. Overall, 33% of respondents reported exposure, with substantially higher prevalence among women (53%) than men (18%). Most incidents involved patients or clients (84%), although colleagues were also frequently identified as perpetrators (57%). Respondents attributed such exposure to both individual and organizational factors, including workplace culture, coarse humor, permissive attitudes, and women’s minority position in a male-dominated work environment [36]. These findings were complemented by Kettunen, who identified broader organizational patterns of inappropriate behavior and discrimination within a rescue department. Nearly half of respondents reported inappropriate behavior, while views on the occurrence of discrimination within the organization varied: 35% perceived discrimination, 40% did not, and 25% were unsure. Overall, 21% reported personal experiences of discrimination, with gender identified as the most common single reason [37].

These quantitative findings were further supported by qualitative data, which provided insight into how gendered exposure to violence and discrimination is experienced in everyday practice. Women EMS professionals described degrading and gendered treatment from male patients, relatives, and colleagues, including verbal comments, questioning of authority, and differential treatment in patient encounters [38].

### Critical incidents, stress, and support-seeking

Evidence regarding critical incidents was derived primarily from one cross-sectional survey, complemented by one qualitative study addressing support-seeking behaviors [38, 39]. All EMS professionals reported exposure to a wide range of critical incidents, with exposure increasing with work experience and being more frequent among men. Men experienced significantly more critical incidents than women, including seeing someone dying (*p* = 0.010), encountering decaying corpses, mutilated bodies, and SIDS (*p* < 0.001), child-related incidents including severe injuries and accidents (*p* < 0.001), severely burned patients (*p* < 0.001), and suicide victims (*p* = 0.020). Women reported significant stress related to critical incidents. However, levels of critical incident-related stress were similar between women and men (mean 20.21 vs. 19.70) [39]. Differences were observed in coping and support-seeking behaviors. Women reported a greater tendency to seek support from healthcare services than men did, while both groups relied most on colleagues for support. Overall, gender differences were observed in exposure to critical incidents and support-seeking behaviors, whereas stress levels and intention to leave the profession showed limited gender variation [39]. Similar findings on support-seeking were reported by Jonson & Lindberg, who found that women EMS professionals primarily sought support from colleagues and personal networks, while support from managers was described as limited or absent [38].

### Professional roles, competence, and organizational conditions

Evidence regarding competence and leadership was limited and originated from a small number of studies addressing different aspects of professional roles [38, 40]. Gender differences were observed in self-assessed competence. Women scored significantly higher in Nursing care (*p* = 0.028) and Value-based Nursing (*p* = 0.047), whereas men reported higher self-assessed competence in Medical Technical Care (*p* = 0.048) and Care Environments in Serious Events (*p* = 0.011) [40]. However, the qualitative study reported that women EMS professionals described recurrent exposure to questioning of competence, being corrected, and needing to repeatedly demonstrate their capability in everyday work situations [38].

Gender differences were evident in leadership representation and career progression. Despite an equal gender distribution among EMS professionals (*n* = 324 women, *n* = 308 men), all EMS field commander positions were held by men (*n* = 32), with only a small number of women occupying substitute roles (*n* = 20) [41]. While men and women reported similar levels of interest in leadership roles, men applied for these positions at approximately twice the rate of women. Among women EMS professionals, longer work experience was significantly associated with applying for leadership positions (*p* = 0.027), whereas no such association was found among male EMS professionals (*p* = 0.174). Neither internal nor external factors were found to influence willingness to apply differently by gender. However, men perceived workplace equality and the presence of double standards more positively than women [41]. Regardless, the qualitative study found that women EMS professionals experienced difficulties being heard within the organization, as well as limited managerial response and a lack of follow-up [38].

## Discussion

This review synthesized gender-related findings across Nordic EMS research. The findings are interpreted through the lens of organizational socialization, which is applied as an interpretive lens rather than the review’s analytical framework. This perspective provides a way to understand how workplace norms, professional identity, and informal socialization processes shape access to legitimacy, role clarity, social integration, and opportunities within EMS organizations [15, 18, 22].

A finding in our review is the disconnect between research aims and reported findings. Less than half of the studies explicitly aimed to investigate gender or gender inequality, yet gender-related patterns were observed. This invisibility of gender as an explicit research area in Nordic countries, despite its empirical presence [10], is unlikely to be entirely a neutral oversight; rather, it suggests that gender may operate as an implicit organizing principle that remains analytically under-articulated [15–17]. Researchers, like clinicians, are socialized into professional contexts where masculine norms function as the unmarked default, rendering gender simultaneously present and unarticulated [15, 22]. This may explain why gender emerged across the included studies despite rarely being treated as an explicit object of investigation.

From an organizational perspective, our findings suggest that EMS professionals may be introduced to a professional culture characterized by expectations of performance, resilience, and emotional control. These expectations appear to shape what is understood as a “competent” EMS professional and may be formed early and reinforced over time within the profession. Previous work has described how EMS professionals are expected to function as “problem solvers,” balancing task performance, role clarity, and social acceptance [1–4, 19, 42, 43]. This is consistent with broader occupational health research suggesting that gendered working environments influence mental health, although the effects vary depending on the specific workplace exposures being examined. Rather than attributing differences solely to biological sex, these studies emphasize the importance of examining organizational contexts through a gender lens [14].

Rather than representing isolated individual gender differences, the findings in this review suggest that organizational processes may shape how workplace experiences, competence, leadership opportunities, and exposure to risk are distributed within EMS. Women reported higher physical and psychosocial demands, greater concerns about violence, poorer mental health outcomes, and higher intention to leave, while simultaneously demonstrating higher physical capacity and similar levels of critical incident stress [30]. The qualitative findings further illustrate how these processes are enacted in everyday interactions, where competence is not only performed but also negotiated in relation to gendered expectations [38]. Rather than reflecting inconsistencies in experience, this indicates that organizational membership is conditional on meeting norms constructed around dominant-group expectations. In such contexts, higher performance may function as a prerequisite for legitimacy rather than an advantage, as suggested by Kanter’s work on tokenism [16]. This reflects a conditionality of belonging, in which inclusion and “fitting in” within the professional community are contingent on aligning with norms of endurance and emotional control. This dynamic may also explain the normalization and underreporting of workplace violence described in our findings [33]. If managing physical threat and psychological strain is embedded within the professional identity, reporting such experiences may constitute a deviation from expected norms. In this sense, underreporting can be understood as a consequence of socialization processes that define endurance as a marker of competence [18, 44]. Similarly, women’s greater tendency to seek formal support [39] may reflect a response to a greater burden, thereby positioning them at the margins of dominant coping norms.

These patterns align closely with Acker’s conceptualization of gendered organizations, where the “ideal worker” is implicitly constructed around disembodied, masculine norms that obscure the conditions under which work is performed [15]. Within EMS, the “heroic” or endurance-based professional ideal may function as such a standard, shaping both expectations and evaluations of competence. Together, organizational socialization and Acker’s perspective help explain how these norms are transmitted and reproduced through everyday practices that reward, tolerate, or mark as deviant those who “stand out” [15, 18, 22]. The findings on leadership and career progression further support this interpretation. Despite equal gender representation among EMS personnel, leadership positions were primarily held by men, and men applied for these roles at higher rates despite similar levels of interest [41]. This may reflect differences in anticipatory socialization and access to social integration, influencing who is perceived, and perceives themselves, as legitimate candidates for leadership [18, 22]. This may suggest that pathways to leadership are not neutral but shaped by underlying norms that advantage those already aligned with dominant organizational expectations [17].

The Nordic setting adds an important layer of complexity. While these societies are often associated with strong commitments to gender equality, formal equality does not necessarily translate into equal experiences in everyday work. Given the limited number of studies included and the relatively small proportion explicitly addressing gender, the lack of focus on gender may partly reflect an assumption that equality has already been achieved, potentially making remaining inequalities less visible in both clinical practice and research. Taken together, the findings suggest that gender-related patterns in Nordic EMS are not isolated observations but appear across multiple aspects of the work environment. Rather than reflecting isolated differences between women and men, these recurring patterns suggest that work demands, recognition of competence, workplace risks, and access to leadership are shaped by organizational norms and informal socialization processes that influence legitimacy and opportunities within EMS. These patterns may indicate how norms and expectations within EMS may influence work experience, access to opportunities, and exposure to risks, with potential implications for both professionals and organizations.

### Strengths and limitations

Despite the relatively limited number of studies identified, this review has strengths. A scoping review methodology provides a structured mapping of the extent and nature of gender-related findings within Nordic EMS research. The inclusion of multiple study designs allowed for a broad overview of workplace conditions, health outcomes, and exposure to violence across different contexts. Furthermore, the focus on secondary gender findings enabled the identification of patterns that might otherwise remain overlooked. However, several limitations should be considered. First, the number of studies included was limited, and fewer than half explicitly focused on gender as a primary research aim. The search strategy did not explicitly include concepts related to masculinities, masculine role norms, or male-dominated workplace cultures. Consequently, studies primarily addressing gendered organizational cultures may not have been identified, which may have influenced the scope of the available evidence. Gender-related findings were also often reported inconsistently and not systematically analyzed, which limits the depth of interpretation. In addition, some findings were based on secondary analyses or subgroup analyses, which may increase the risk of reporting bias and limit the robustness of gender-related conclusions. Second, the heterogeneity of study designs, measures, and outcomes limits comparability across studies. Several studies used survey designs with relatively low response rates, which may introduce non-response bias and affect the representativeness of the findings. Third, the methodological quality of the included studies varied, and most were observational or descriptive, limiting the depth and consistency of the available evidence. Fourth, based on our findings, it was not possible to examine underlying mechanisms or causal relationships. Finally, several findings related to discrimination, harassment, equality and leadership originated from master’s theses. Their inclusion broadened the evidence base in this under-researched field, although these studies had undergone academic examination at university level rather than external peer review.

## Conclusion

This scoping review found that gender-related patterns are present across Nordic EMS research but are rarely examined as a primary focus. Rather than representing isolated findings, these patterns reflect underlying organizational processes through which norms, expectations, and professional identities are shaped and reproduced. Interpreted through an organizational socialization perspective, the findings suggest that access to a sense of belonging, legitimacy, and career opportunities may be unevenly distributed, with implications for EMS professionals’ well-being, exposure to workplace risks, and workforce sustainability. Therefore, EMS organizations should consider how workplace norms, onboarding, leadership development, mentoring, and organizational support systems influence opportunities for participation, recognition, and professional development. Addressing these organizational processes may contribute to more inclusive workplaces, improved staff well-being, and a more sustainable EMS workforce. The findings highlight the need for future research to more explicitly address gender within Nordic EMS contexts, particularly by examining how organizational practices, socialization processes, and workplace cultures contribute to differential experiences and outcomes. Such insights are essential for developing more inclusive, equitable, and sustainable Nordic EMS organizations.

## Supplementary Information

Below is the link to the electronic supplementary material.

Supplementary Material 1

Supplementary Material 2

## Data Availability

No datasets were generated or analysed during the current study.
