# Supplementary material for: Fitting in or standing out: a scoping review of gender-related findings in Nordic EMS through an organizational socialization lens
Source: Scand J Trauma Resusc Emerg Med. 2026 Aug 17;34:139. doi: 10.1186/s13049-026-01677-3 (PMC13495196; doi:10.1186/s13049-026-01677-3)
Supplement: Supplementary file 2 [file 13049_2026_1677_MOESM2_ESM.docx]

**Supplementary file.**

Included studies in the scoping review: *Fitting in or standing out: A scoping review of gender discrepancies in Nordic EMS through an organizational socialization lens*, Lindström, V. et al. (2026)

| **Author, Year, Country** | **Aim(s)** | **Study design** | **Population and sample** | **Main Findings** |
| --- | --- | --- | --- | --- |
| Jansson et al. (2020) Sweden | To investigate and compare self-reported professional competence among nurses working in the ambulance service and to explore associations between potentially predictive background factors and self-reported professional competence. | Cross-sectional | 3 ambulance organizations in central Sweden. 105 nurses in the ambulance service participated in a questionnaire, with a response rate of 21%. | The findings showed that being a woman was positively associated with higher competence in Nursing care and Value-based Nursing. In Medical Technical Care and Care Environments Serious Events, men reported significantly higher self-reported competence than women. |
| Johnsen et al. (2024), Sweden | To describe work-, lifestyle-, and health-related factors among ambulance personnel, and to analyse differences between women and men. | Cross-sectional | 106 EMS clinicians self-reported and objective measures of work, lifestyle, and health in 10 Swedish ambulance stations | Women reported higher physical work demands compared to men. They also reported working more frequently in awkward or bending positions and experiencing higher psychosocial work demands. Women more frequently reported concerns about threats and violence compared to men, and a higher proportion also reported having been subjected to threats. Women reported higher levels of mental health problems compared to men, and a greater proportion also reported sleep disturbances and worry. Women performed better in several physical capacity tests. Men had higher levels of cardiovascular disease risk factors, while women demonstrated a lower risk of cardiovascular disease. |
| Juliusdatter Haug et al. (2025), Norway | To adapt and validate the Staff Observation Aggression Scale – Revised (SOAS‑R) for use in ambulance services (SOAS‑RA), and to examine the relationship between SOAS‑RA severity scores and staff’s subjective perceptions of incident severity using a Visual Analog Scale (VAS). | Delphi | 402 reports from EMS (Emergency Medical Service) clinicians, 34 ambulance stations. | Women rated incidents of workplace violence as more severe than men, despite no differences in objectively measured severity scores, indicating potential gender differences in perception and appraisal of workplace threats. |
| Kettunen, M. (2022), Finland (Master's thesis) | The purpose of the thesis was to examine the current state of equality in the North Karelia Rescue Service and prepare an equality plan for them. | Multi-method | 1 rescue department. Participants: n=174, male n=139 (80 %), female n=22 (13 %). Paramedics: 34 % (n=59) | Inappropriate behavior was perceived to occur within the rescue department by 47% of respondents. The most reported individual reasons were age (24% and gender (21%). Regarding discrimination, 35% of respondents reported that it occurs within the rescue department, while 40% felt that it does not occur. 21% of respondents reported having personally experienced discrimination. Gender was identified as the most common single reason for discrimination (27%). |
| Koskela A. (2025), Finland, (Master's Thesis) | To analyze the relationships among background variables, internal factors (confidence, expected traits of a leader), external factors (perceived equality, double standards, influence of parenthood), and paramedics’ perceptions of becoming an EMS field commander. | Cross-sectional | Paramedics in 4 Finnish rescue departments providing EMS and EMS field management (n = 632). | Despite equal representation of women among EMS clinicians (n = 324 women, n = 308 men), all EMS field commanders are men (n = 32), with only five women occupying substitute roles. Internal and external factors did not influence the willingness to apply differently by gender. Men viewed equality and double standards more positively than women did. Among women, there was a significant relationship between longer work experience and applying for the EMS field commander position. For male paramedics, no significant relationship was found. |
| Lindberg, J. (2025) Sweden (Master's Thesis) | The purpose of the study was to highlight female ambulance nurses’ experiences of harassment in their professional roles. | Qualitative | 8 female ambulance nurses | Participants described a workplace culture characterized by power imbalances and gendered norms, where they were undermined, questioned, and exposed to degrading behavior from male colleagues, patients, and relatives. These experiences had a mental impact, including reduced self-esteem, emotional distress, and difficulties maintaining professional composure across patient encounters. The participants described a continuous need to prove their competence and legitimacy, often by overperforming or accepting unfavourable conditions. Organizational participants reported a lack of managerial support, characterized by a culture of silence, insufficient action on reported incidents, and limited competence in handling gender-related issues. Responsibility for managing discrimination was often perceived as being shifted onto the individual. As a result, women primarily relied on informal support systems, particularly colleagues and personal networks, rather than formal organizational structures. |
| Luoma, K. (2020) Finland (Master's thesis) | The aim of the work was to identify the current state of experiences in equality and to promote equality through behavioral economics in the field of rescue services. | Multi-method | 11 rescue services (including paramedics) in Finland. Participants: n=337, response rate 15 %. | Fewer than 23% of all respondents reported personally experiencing gender discrimination; the burden was strongly concentrated among women: nearly 69% of women had experienced gender-based discrimination, and as many as 85% had observed it. The burden was most pronounced in EMS, where younger women at the beginning of their careers appeared especially vulnerable, whereas discrimination was reported less often in expert and accident prevention roles with more balanced gender representation. |
| Nordqvist et al. (2023) Finland | To examine the prevalence of critical incident (CI) experiences, critical incident stress (CI-S), and CI-S association with the intention to leave the paramedic profession, as well as the needed and received support for CI-S among Finnish paramedics. | Cross-sectional | 427 Paramedics from 8 ambulance organizations answered a survey. | All paramedics reported exposure to CI, with experiences increasing with work experience, particularly among men. Men reported more frequent exposure to several types of CI. Levels of CI-stress were similar between women and men. Women reported a greater tendency to seek support from healthcare services than men, while both groups most commonly relied on colleagues. |
| Petzäll et al. (2011), Sweden | To investigate the incidents of threats and violence within the Swedish ambulance service and to describe these situations | Cross-sectional | 134 registered nurses and paramedics from 11 ambulance stations answered a survey. | Most EMS clinicians reported exposure to threats and violence. Women reported higher rates of both threats and physical violence compared to men, although these differences were not statistically significant. Threats were most often verbal threats of physical violence. Physical violence typically consisted of pushing, hitting, and kicking. In most cases, the perpetrator was the patient, often under the influence of alcohol or drugs. Exposure to threats and violence led to behavioral changes. |
| Schøsler et al. (2024), Denmark | To describe the extent of and subsequent reporting of physical and psychological workplace violence toward the prehospital healthcare workers in Denmark in a two-year period. Moreover, we wanted to elucidate any possible effect of workplace violence on the private and professional lives of the prehospital healthcare personnel. | Cross-sectional | 584 prehospital health care workers answered a survey from different parts of Denmark | In a nationwide survey, 47.4% of EMS clinicians reported exposure to psychological violence. The perpetrators were primarily patients or relatives, and physical violence was most involved in pushing, punching, and kicking, while psychological violence included threats and intimidation. Exposure to violence was associated with both physical and psychological consequences, including stress, anxiety, depression, and sleep disturbances. Violence was frequently underreported, with 60.7% not reporting physical violence and 83.0% not reporting psychological violence to their employer. The most common reasons for not reporting were that incidents were perceived as not serious enough or that reporting was unlikely to make a difference. |
| Viking et al. (2024) Sweden | To measure the 1-year cumulative incidence of and analyze the risk factors associated with workplace violence directed towards the ambulance service in a Swedish region | Prospective cohort study | 28 640 ambulance assignments in one region in Sweden | Most EMS clinicians reported exposure to threats and violence, with 66% having experienced it. Women reported higher rates of both threats and physical violence compared to men, although these differences were not statistically significant. |
| Ylönen, J. (2019), Finland (Master thesis) | The aim of the study was to find out how common the sexual harassment experienced by the primary care community in the Central Rescue Department of Finland is, and what factors expose paramedics and students to sexual harassment. | Cross-sectional | 1 rescue department and 1 University of Applied Sciences. Target groups: rescue workers, paramedics, and paramedic students. Participants: n=99, response rate 24 %. | 33% of the respondents had experienced sexual harassment at work or during an internship. 53% of the women, and 18% of the men. The most sexual harassment cases reported had been caused by patients or clients (84%), but sexual harassment was also reported to have been caused by colleagues (57%). In 94% of the cases of sexual harassment, the harassers were of a different sex than the subjects of that harassment. Exposure to sexual harassment was, in most cases, promoted by one’s own sexually provocative behavior, the attitude of the work community, coarse humor, and the presence of female colleagues in a predominantly male work community. Several forms of avoidance of sexual harassment in the work community were detected such as training, and development of the work community, an open climate for talking about sexual harassment, and physical architecture. |
